# Metagenomic sequencing of human cardiac tissue reveals Microbial RNA which correlates with Toll-like receptor-associated inflammation in patients with heart disease

**DOI:** 10.1038/s41598-023-35157-w

**Published:** 2023-05-15

**Authors:** Joakim Sandstedt, Kristina Vukusic, Göran Dellgren, Anders Jeppsson, Lillemor Mattsson Hultén, Victoria Rotter Sopasakis

**Affiliations:** 1 Department of Clinical Chemistry, Sahlgrenska University Hospital, and Department of Laboratory Medicine, Institute of Biomedicine, Sahlgrenska Academy, University of Gothenburg, Gothenburg, Sweden; 2 Department of Cardiothoracic Surgery, Sahlgrenska University Hospital, Gothenburg, Sweden; 3 Department of Molecular and Clinical Medicine, Institute of Medicine, Sahlgrenska Academy, University of Gothenburg, Gothenburg, Sweden; 4 Wallenberg Laboratory, Department of Molecular and Clinical Medicine, Institute of Medicine, Sahlgrenska Academy, University of Gothenburg, Gothenburg, Sweden

**Keywords:** Genetics, Molecular biology, Systems biology, Cardiology, Molecular medicine

## Abstract

Cardiovascular disease (CVD) is strongly associated with chronic low-grade inflammation, involving activated Toll-like receptors and their downstream cellular machinery. Moreover, CVD and other related inflammatory conditions are associated with infiltration of bacteria and viruses originating from distant body sites. Thus, in this study we aimed to map the presence of microbes in the myocardium of patients with heart disease that we previously found to display upregulated Toll-like receptor signaling. We performed metagenomics analysis of atrial cardiac tissue from patients undergoing coronary artery bypass grafting (CABG) or aortic valve replacement (AVR) and compared with atrial cardiac tissue from organ donors. A total of 119 species of bacteria and seven species of virus were detected in the cardiac tissue. RNA expression of five bacterial species were increased in the patient group of which *L*. *kefiranofaciens* correlated positively with cardiac Toll-like receptor-associated inflammation. Interaction network analysis revealed four main gene set clusters involving cell growth and proliferation, Notch signaling, G protein signaling and cell communication in association with *L.* *kefiranofaciens* RNA expression. Taken together, intracardial expression of *L*. *kefiranofaciens* RNA correlates with pro-inflammatory markers in the diseased cardiac atrium and may have an effect on specific signaling processes important for cell growth, proliferation and cell communication.

## Introduction

Cardiovascular disease (CVD) is strongly associated with a chronically elevated inflammatory state, involving pro-inflammatory cytokine production following upscaled Toll-like receptor (TLR) action, triggered by various factors, such as pathogen-associated microbial patterns (PAMPs) and damage-associated molecular patterns (DAMPs), stress, hypoxia and ischemia^1–3^. Loss-of-function studies where TLRs are targeted show that a sustained inflammation is harmful to the heart and contributes to cardiac adverse remodeling^4,5^, which clinically is manifested as changes in size, mass and function of the heart^6^.

We have previously reported increased expression of TLRs 1, 3 and 7 and their mediators in the cardiac tissue of patients undergoing coronary artery bypass grafting (CABG) or aortic valve replacement (AVR)^7^, suggesting that triggers of viral/microbial origin might be involved in the augmented cardiac inflammatory state in these patients; TLRs 1, 3, and 7 are associated with viral infections and recognize viral single-stranded and double-stranded RNA as well as PAMPs from gram positive bacteria^8–10^.

CVD and other related pathological conditions, such as diabetes, obesity and non-alcoholic fatty liver disease, have been associated with, and shown to be modified by, bacteria and viruses, originating from distant body sites, such as the oral cavity. For example, in a large study with close to 12,000 participants, poor oral hygiene was associated with increased risk of fatal and non-fatal CVD events, including myocardial infarction, coronary artery bypass graft surgery, coronary angioplasty, stroke, heart failure and low-grade inflammation^11^. Altered microbial composition of the gut has also been associated with symptomatic atherosclerosis, i.e. myocardial infarction or cerebrovascular events^12^, and certain groups of bacteria have been shown to predict coronary artery disease^13^. Furthermore, the presence of bacterial DNA in human atherosclerotic plaques has been demonstrated in several reports involving different patient groups and various techniques (reviewed in ref.^14^). Thus, there is a large body of support for bacterial and viral expression in human tissue that is associated with low-grade inflammation and cardiovascular disease.

In this study we used metagenomics to investigate microbial gene expression in human myocardium with elevated inflammation from patients undergoing CABG or AVR and compared with normal myocardium.

## Results

We performed metagenomics analysis of microbial RNA expression on right atrial tissue collected during coronary artery bypass grafting (CABG) and aortic valve replacement (AVR). A comparison was made with tissue from a control group of right atrium harvested from multi-organ donors without cardiac disease as well as commercially available right atria RNA from healthy individuals. A summary of the patient clinical data is provided in Tables 1 and 2.

**Table 1:** Clinical data summary.

	Controls (n = 10)	CABG patients (n = 10)	AVR patients (n = 10)
Age, mean ± SD	47 ± 20.5	64 ± 8.2	73.1 ± 6.4
Sex (M/F)	5/5	9/1	7/3
BMI		26.2 ± 1.8	28.8 ± 4.4
Hypertension		5	7
Aortic stenosis		0	10
Atrial fibrillation		1	1
Aortic regurgitation		0	6
Angina pectoris		7	0
Pathologic Q wave		2	0
Poor R wave progression		1	0
Suspected LVH		1	0
Endocarditis		0	1
Myocardial infarction		2	0
Hyperlipidemia		1	0
Smoking		2	1
Type 2 diabetes		1	1

**Table 2:** Clinical background of the organ donor controls.

Donor	Sex	Age	Cause of death	Other diseases
1	F	50	Intracerebral hemorrhage	Previous ventricular tachycardia, suspected previous AMI, suspected Takotsubo cardiomyopathy
2	M	51	Ischemic cerebral edema due to cardiac arrest	HF in the acute setting
3	F	19	Ischemic cerebral edema due to cardiac arrest	Anorexia
4	F	43	Ischemic cerebral edema due to cardiac arrest caused by major bleeding	None
5	M	52	Cardiac arrest	HF in the acute setting

*F* female, *M* male, *AMI* acute myocardial infarction, *HF* heart failure.

**Figure 1:**
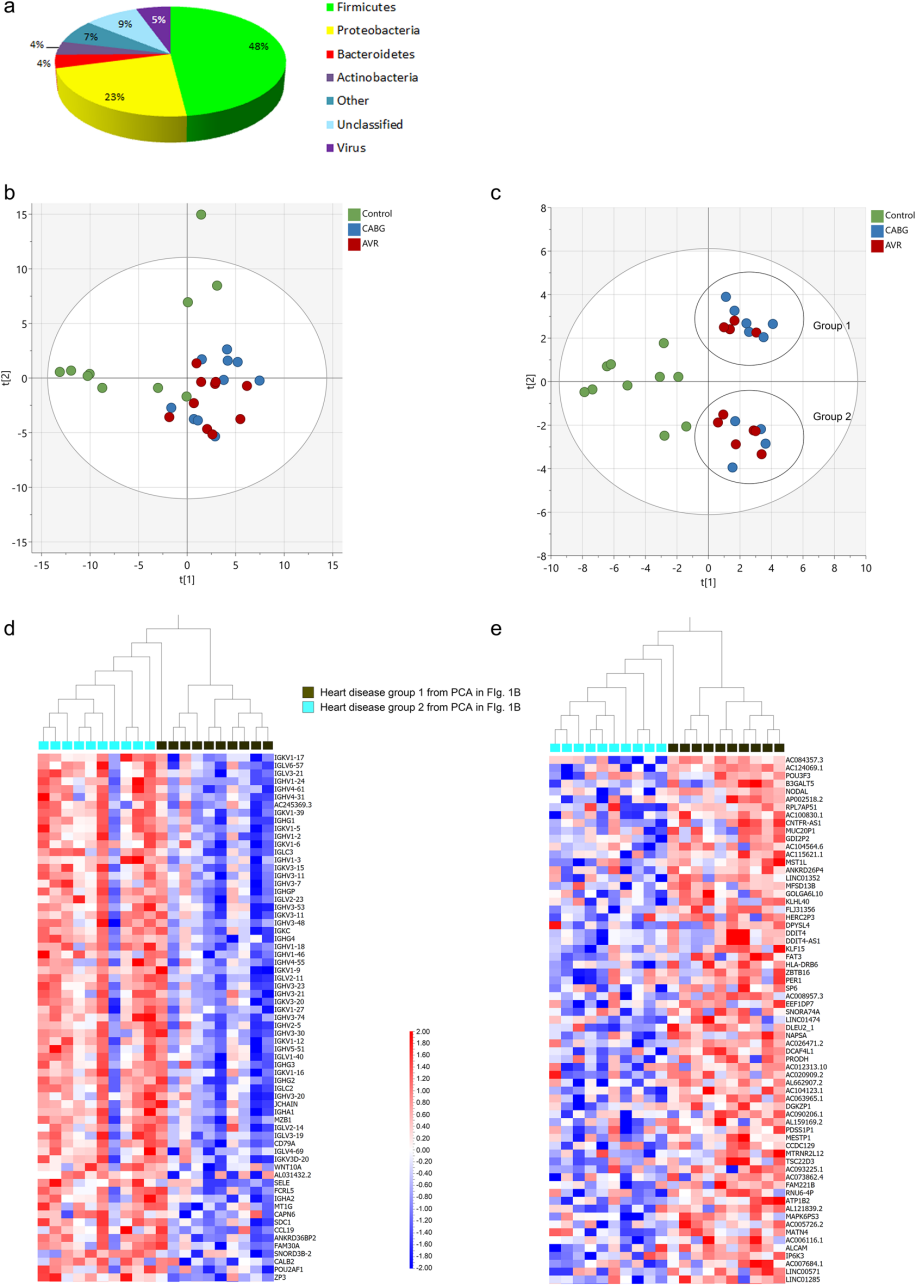
Cardiac microbial RNA analysis. (**a**) Proportion of microbial organisms and bacterial phyla detected in human atrial tissue, (**b**) PCA score plot showing a separation between patients with heart disease (CABG and AVR) and controls with regard to microbial RNA expression in the atrial tissue, (**c**) PCA score plot of microbial RNA expression significantly different between the controls and patients with heart disease in atrial tissue showing a separation of two subgroups (designated group 1 and group 2) within the patient group, (**d**–**e**) heat map of genes most significantly decreased (**d**) or increased (**e**) in group 2 versus group 1 in (**c**) (log_2_ fold change, q-value < 0.05). Heatmaps were generated using the Qlucore Omics Explorer 3.8 software (Qlucore AB, Lund, Sweden, https://qlucore.com/). *n* = 10 for CABG patients, 10 for AVR patients and 10 for controls.

A total of 119 species of bacteria and seven species of virus were detected in the cardiac tissue (Supplementary Table 1). The majority of the bacteria was classified as Firmicutes (48%), followed by Proteobacteria (23%), Bacteroidetes (4%) and Actinobacteria (4%) (Fig. 1a). Using Principal Component Analysis (PCA), an unbiased multivariate classification model, we observed a cluster separation between the control individuals and the patients with heart disease with regard to microbial gene expression (Fig. 1b). There was no clear separation between the CABG and AVR groups (Fig. 1b). A similar pattern was observed when only including microbial expression that was significantly different between the controls and patients with heart disease (Fig. 1c). Although no separation between the CABG and AVR groups was observed, there was a distinct separation within the group of individuals with heart disease (Fig. 1c). This separation could not be explained by a difference in gender, age or BMI of the individuals included (data not shown), and thus we performed bioinformatic analysis with regard to gene expression in these two groups. The result showed that the two groups differed in gene expression of genes encoding antibody light- and heavy chains (Fig. 1d) as well as various long non-coding RNAs (Fig. 1e). GSEA revealed an association of these genes with mast cell activation, phagocytosis and complement activation, particularly molecular processes involving high affinity receptor for the Fc region on immunoglobin E (FCERI) action, signaling through phosphoinositide 3-kinase (PI3K) as well as intracellular calcium signaling (Table 3 and Supplementary Fig. 1).

**Table 3:** Signaling pathways differing between heart disease subgroups 1 and 2 following PCA.

GSEA reactome pathway name	NES
Role of phospholipids in phagocytosis	2.00
FCERI-mediated NFkB activation	1.95
Creation of C4 and C2 activators	1.95
Binding and uptake of ligands by scavenger receptors	1.92
Initial triggering of complement	1.92
FCERI-mediated Ca^2+^ mobilization	1.91
Role of LAT2, NTAL and LAB on calcium mobilization	1.91
FCERI-mediated MAPK activation	1.90
Scavenging HEME from plasma	1.89
Complement cascade	1.89
Chr2p11	1.88
Antigen activates B cell receptor (BCR) leading to generation of second messengers	1.86
Regulation of actin dynamics for phagocyte formation	1.86
Fc epsilon receptor FCERI signaling	1.85
Cell surface interaction at the vascular wall	1.85
FCGR activation	1.84
Fc gamma receptor FCGR-dependent phagocytosis	1.81

*NES* normalized enrichment score.

Taken together, metagenomics analysis revealed microbial RNA expression in the heart tissue of both controls and patients with heart disease. Subsequent PCA showed an apparent separation between the groups with regard to the cardiac microbial RNA expression. Furthermore, we observed two distinct subgroups within the group with heart disease (CABG and AVR combined) that displayed genetic differences related to mast cell activation, phagocytosis and complement activation.

### RNA expression from five bacterial species is significantly increased in diseased myocardium

**Figure 2:**
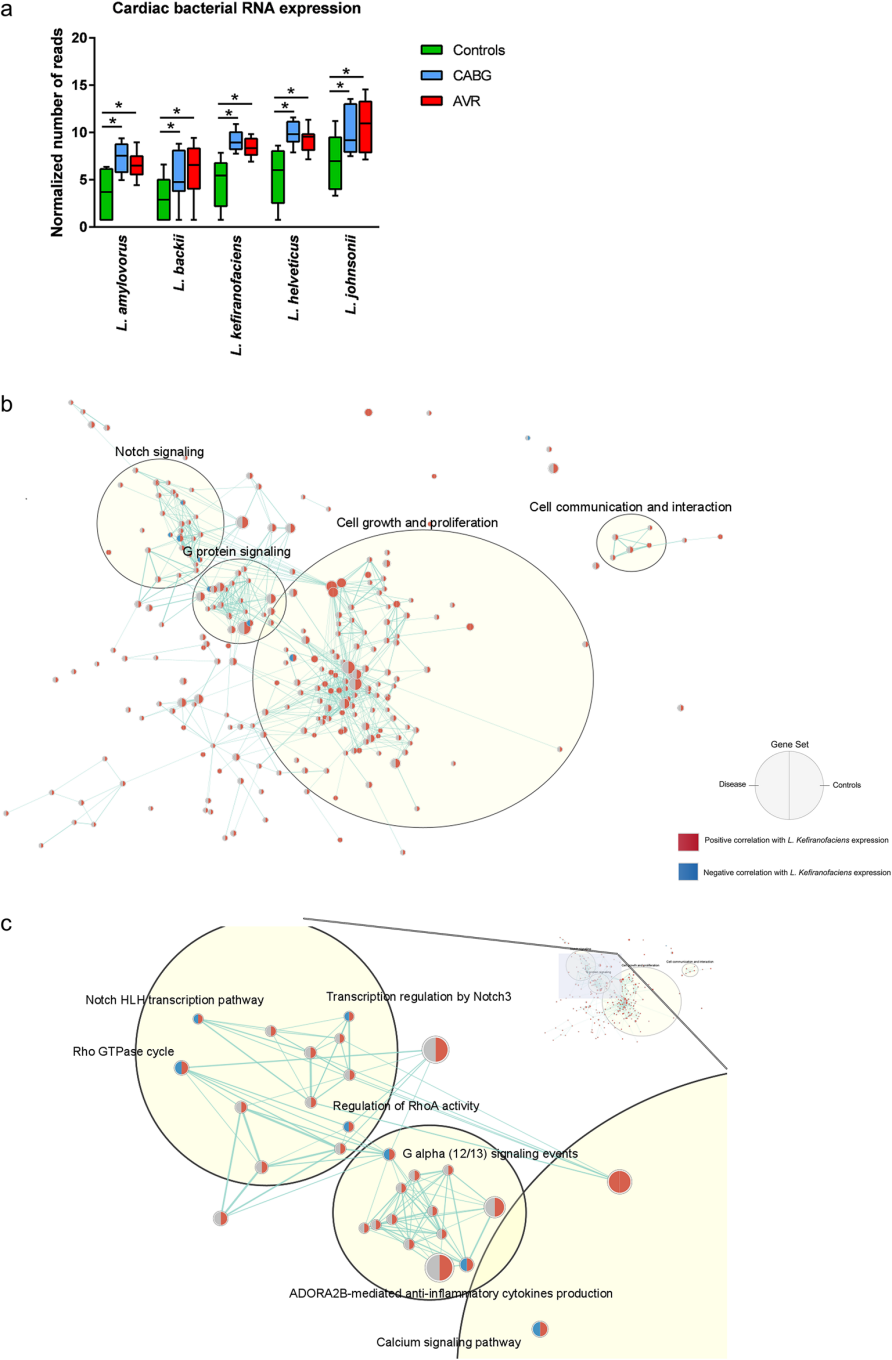
Microbial RNA expression analysis in patients with heart disease compared to controls. (**a**) Bar graph displaying cardiac atrial RNA expression of *Lactobacillus amylovorus, Lactobacillus backii, Lactobacillus kefiranofaciens, Lactobacillus helveticus and Lactobacillus johnsonii* in controls, CABG patients and AVR patients. Error bars indicate SEM, *p < 0.05. (**b**,**c**) Correlation analysis between cardiac *L. kefiranofaciens* expression and human gene sets in patients with heart disease (CABG and AVR combined) and controls through Gene Set Enrichment Analysis (GSEA) of RNA seq-data from cardiac atrial tissue followed by correlation analysis with *L*. *kefiranofaciens* expression in the same individuals. (**b**), Interaction network analysis revealed four main gene set clusters that correlated with *L*. *kefiranofaciens* expression in controls: cell growth and proliferation, Notch signaling, G protein signaling and cell communication and interaction. (**c**), Close-up of seven gene sets that displayed opposite correlation patterns for controls compared to patients with regard to *L*. *kefiranofaciens* expression. Nodes = gene sets. Red color within the gene set nodes indicate positive correlation of those gene sets with cardiac atrial expression of *L. kefiranofaciens*, blue color within the gene set nodes indicate negative correlation, grey color within the gene set nodes indicate no significant correlation. The drawings were generated using the Cytoscape v. 3.9.1 software and the EnrichmentMap app (https://cytoscape.org). *n* = 10 for CABG patients, 10 for AVR patients and 10 for controls.

Among the microbial expression that was significantly increased in the diseased myocardium compared to controls, the bacterial species with highest overall alignment rate percentage, ranging from 76 to 92%, were classified as *Lactobacillus amylovorus, Lactobacillus backii, Lactobacillus kefiranofaciens, Lactobacillus helveticus and Lactobacillus johnsonii* (Fig. 2a). The reads were evenly spread over the genomes for *L. amylovorus, L. kefiranofaciens*, *L. helveticus* and *L. johnsonii*, indicating a good fit to the chosen reference genome (data not shown). The reads for *L. backii* were more focused to a few specific regions.

### *L*.* kefiranofaciens* shows strong correlation to cardiac inflammatory markers associated with Toll-like receptor signaling

We have previously reported an increased inflammatory state in the cardiac tissue from five of the patients included in the current cohort^7^ and were therefore interested in investigating if there is a correlation between the expression of the assigned bacterial species and cardiac inflammatory markers associated with Toll-like receptor (TLR) signaling. Of the five bacterial species with increased RNA in the patients with cardiac disease, *L. kefiranofaciens* positively correlated with 31 TLR-induced inflammatory mediators (Table 4 and Supplementary Fig. 2), whereas *L. amylovorus*, *L. helveticus*, *L. backii* and *L. johnsonii* did not correlate with any of the inflammatory markers investigated (data not shown).

**Table 4:** Linear regression analysis of Toll-like receptor-associated inflammatory markers and cardiac atrial *L*. *kefiranofaciens* RNA expression in controls (n = 4), CABG- (n = 5) and AVR (n = 5) -patients (combined).

*L*. *kefiranofaciens*			
Gene	Type of correlation	FDR adjusted p-value	R^2^ value
IRAK1	Positive	0.008	0.63
TIRAP	Positive	0.012	0.54
SARM	Positive	0.013	0.52
TNFRSF1A	Positive	0.014	0.50
HRAS	Positive	0.015	0.50
NFKB2	Positive	0.017	0.46
CHUK	Positive	0.018	0.46
TICAM1	Positive	0.018	0.45
PTGS2	Positive	0.019	0.44
TOLLIP	Positive	0.020	0.43
UBE2N	Positive	0.023	0.41
FADD	Positive	0.024	0.40
RELA	Positive	0.027	0.38
IRF1	Positive	0.027	0.38
PRKRA	Positive	0.029	0.38
PPARA	Positive	0.032	0.35
NFKB1	Positive	0.033	0.34
ELK1	Positive	0.034	0.34
FOS	Positive	0.036	0.33
MAP2K4	Positive	0.037	0.32
CD14	Positive	0.039	0.32
REL	Positive	0.041	0.31
MAPK8IP3	Positive	0.042	0.30
MAP4K4	Positive	0.043	0.30
MAP3K1	Positive	0.045	0.29
ECSIT	Positive	0.045	0.29
SIGIRR	Positive	0.046	0.29
CD180	Positive	0.047	0.29
IL1B	Positive	0.048	0.28
JUN	Positive	0.049	0.28
CCL2	Positive	0.049	0.28

Plasma from the patient cohort was analyzed, using a high-throughput, multiplex immunoassay enabling analysis of 92 human cardiovascular and inflammatory biomarkers. None of the plasma CVD biomarkers from the multiplex panel correlated with the cardiac RNA expression of the observed bacterial species (Supplementary Table 2).

Taken together, out of the five bacterial species whose expression was most significantly upregulated in the patients with heart disease (CABG and AVR combined), *L. kefiranofaciens* had the strongest correlation with TLR-associated intracardiac inflammatory markers. Moreover, the correlation with inflammatory markers seemed confined to the cardiac tissue as no cogent correlation between intracardiac bacterial expression and CVD markers in plasma could be deduced.

### *L*. *kefiranofaciens* expression is associated with transcriptional regulation by Notch, Rho activity, cytokine production and calcium signaling, and may affect these processes differently in individuals with heart disease

Based on the data showing strong correlation between *L. kefiranofaciens* expression and TLR-induced markers, GSEA analysis was performed to investigate possible correlation with *L. kefiranofaciens* expression and other signaling pathways. Interaction network analysis revealed four main gene set clusters involving cell growth and proliferation, Notch signaling, G protein signaling and cell communication and interaction in association with *L. kefiranofaciens* RNA expression (Fig. 2b). The majority of the nodes (gene sets) within these clusters showed a positive correlation (red color) with intracardiac *L. kefiranofaciens* expression in the control individuals whereas no significant correlation (grey color) was found for the patients with heart disease (CABG and AVR combined). Seven gene sets showed opposite correlation patterns for controls vs patients, where *L. kefiranofaciens* RNA expression in the patients was negatively correlated (blue color) with the gene sets and positively correlated in the control tissue (Fig. 2c). These gene sets and their closest neighbor gene sets included processes of transcriptional regulation by Notch, Rho GTPase activity, anti-inflammatory cytokine production and calcium signaling (Fig. 2c).

Taken together, intracardial expression of *L. kefiranofaciens* may have an effect on specific signaling processes important for cell growth, proliferation and cell communication of which some are differentially correlated with this bacterial species in patients with heart disease compared to controls.

## Discussion

In the study presented here we sought to investigate the presence of microbial RNA in the cardiac tissue of patients undergoing CABG- or AVR- surgery and compare with tissue from individuals without heart disease.

The initial Principal Component Analysis (PCA) revealed a separation between the control individuals and the patients with heart disease with regard to microbial gene expression, without a clear separation between the CABG and AVR groups. However, there was a distinct separation within the group of individuals with heart disease that differed with respect to expression of genes encoding antibody light- and heavy chains associated with mast cell activation, phagocytosis and complement activation. It is plausible that the upregulated cardiac expression of antibodies associated with these cellular processes is impacted by specific pathogens within the cardiac tissue. However, that would need to be confirmed with more in depth mechanistic studies and is outside the scope of this report.

Dysbiosis of the intestinal or oral microbiota have long been known to be associated with increased risk of cardiovascular events, such as stroke, myocardial infarction, heart failure and atherosclerosis^14–16^. Such findings are strongly associated with an activated immune system, cytokine levels and other bacterial pro-inflammatory components, such as lipopolysaccharide (LPS) and trimethylamine oxide (TMAO), as well as increased levels of antibodies against bacterial antigens^16–20^. Besides the intestine and oral cavity, other tissues as well as the blood have been reported to harbor microbes in both healthy individuals and disease states^21,22^. The detection of bacteria in disparate tissue sites and blood, including bacterial DNA and RNA as well as various bacterial species, also correlate with CVD in humans^23–25^. The way that bacteria end up in distal organs and tissues following dysbiosis is thought to involve “leakage” through a compromised gut or oral epithelium where bacteria enter the blood stream and cause bacteremia. A well known example of this is endocarditis, where bacterial strains originating from the oral cavity or the gut, leak into the blood stream following inflammation and damage of the supporting tissues of the the teeth and intestinal wall, resulting in infection of the valves^26^. Only one of the patients in our cohort was diagnosed with endocarditis and thus endocarditis can not explain the presence of cardiac bacterial RNA in our study cohort. In addition, bacterial RNA was found also in the control group and the species found were atypical for those normally observed in cases with endocarditis.

We found RNA from 119 bacterial species and seven viral species in the cardiac tissue. RNA from five lactobacilli species was found to be increased in the diseased myocardium compared to control tissue. Additional bacterial strains or viruses might be increased in the diseased heart, but we chose to set strict cut offs and focused on the five bacterial species with the highest overall alignment rate percentage in the bioinformatics analysis.

Ziebolz et al. investigated the presence of DNA of 11 periodontal pathogens in the atrium and ventricle myocardium of 30 patients undergoing aortic valve surgery and detected bacterial DNA in both regions, with a prevalence ranging from 3 to 27% of the patients, depending on the strain investigated^27^. Although the study differs in several aspects from our study (93% of patients had periodontal disease, no healthy controls were included, and only 11 oral pathogens were investigated rather than a full metagenomics analysis), it supports our finding of bacterial nucleic acids in human cardiac tissue.

The bacterial species detected in our study were present also in the heart tissue of the controls. Previously reported data have suggested that the impact of infection on atherosclerosis is related to the total number of pathogens infecting an individual, referred to as pathogen burden^28,29^. In line with this, Koren et al. observed a correlation between the amount of bacterial DNA in atherosclerotic plaques and the leukocyte counts, suggesting that the atherosclerotic plaque bacterial load determined its inflammatory status^25^. In addition, Zhu et al. reported that pathogen burden significantly predicted the combined end point of myocardial infarction or death independent of C-reactive protein in 890 patients with coronary artery disease^30^. Furthermore, the abundance of gut microorgramisms, such Lactobacillales, has been shown to predict coronary artery disease^13^, and the number of Lactobacillales and the ratio of Firmicutes to Bacteroidetes increased in this patient group compared to controls^31^.

In light of this, we found that *L. kefiranofaciens* expression correlated positively with a panel of pro-inflammatory markers in the cardiac tissue in our cohort and was negatively correlated with Notch signaling, Rho GTPase activity, anti-inflammatory cytokine production and calcium signaling in the patient group (CABG and AVR combined), but positively correlated with these pathways in the control group. This suggests that *L. kefiranofaciens* might affect cardiac molecular signaling differently, depending on the amount of bacteria residing in the cardiac tissue.

The *L. kefiranofaciens* species are Gram-positive, non-motile, non-spore-forming, facultative anaerobic rod-shaped lactic acid bacteria known to produce the polysaccharide kefiran, which has been shown to exhibit antimicrobial, immunomodulating and anti-hypertensive properties^32–35^, and is therefore considered a probiotic. However, Lin et al. showed that direct treatment with the *L. kefiranofaciens* subspecies M1 induces inflammation in vitro by upregulating pro-inflammatory cytokines, such as tumor necrosis factor alpha (TNFα), and in vivo by upregulation of proinflammatory cytokines and macrophage markers in mice fed a high fat diet^36^. The discrepancy between different reports regarding the effects of *L. kefiranofaciens* are likely due to different subspecies being investigated, different celltypes/tissues studied as well as underlying pathological conditions. In support of this theory, although many lactobacilli in general are considered probiotics, this genus has been associated with contrasting outcomes when evaluated in CVD animal models, possibly due to strain-specific effects^14,37^. In addition, a recent survey assessing the pathogenic potential of lactobacilli, based on infection case reports, reported that serious infections caused by lactobacilli species normally considered probiotics appear to have increased in the last years. The authors concluded that pathogenic genetic traits, such as biofilm-forming capacity, should be periodically re-evaluated by genetic characterization of strains to identify non-pathogenic variants^38^. With regard to our study cohort, we hypothesize that a possible involvement of *L. kefiranofaciens* in TLR-associated inflammation would occur at a local level, within the human cardiac tissue, as we do not find a salient correlation with levels of proinflammatory markers in the plasma from the same the individuals.

Some limitations of the present study should be acknowledged. The number of individuals in our study is small and the data thus need to be confirmed in larger cohorts. Metagenomics of cardiac tissue in cohorts with other types of heart disease should be investigated as well as different locations of the human heart to put together a more extensive picture of possible intrinsic cardiac microbes in health and disease. Furthermore, it was not possible to match the individuals in the current study for gender and age, both of which could plausibly have an impact on the cardiac inflammatory state. Circulating levels of CRP and IL-6 increase with age and certain sex hormones in serum have been associated with inflammatory biomarkers^39,40^. Of note however, we did not find differences in circulating inflammatory markers between controls and the CABG- or AVR- patients in our previous study using the same cohort as investigated in the current study^7^.

In addition, it must be assumed that many other factors play a role regarding an interplay between cardiac microbes and inflammatory processes, such as environmental and genetic factors. Based on the current study, it is thus not possible to determine whether the observed correlations between bacteria and gene expressions are causal. Although it seems unlikely that for example metabolic syndrome has been an important confounding factor (included study subjects were not obese, only two individuals had type 2 diabetes and we have previously shown that this cohort does not show elevated circulating levels of pro-inflammatory markers^7^), this does not rule out the possibility that other confounder factors may exits. Further studies are thus needed to establish whether there is a causal relationship between bacteria and gene expressions.

In summary, we have identified RNA from five different lactobacilli species in human atrial tissue that was significantly increased in patients with heart disease. *L. kefiranofaciens* RNA expression was associated with cardiac TLR-induced inflammation, and negatively correlated with Notch signaling, Rho GTPase activity, anti-inflammatory cytokine production and calcium signaling in the patient group and may have an effect on specific signaling processes important for cell growth, proliferation and cell communication.

Whether an intrinsic “intracardial microbiota” exists or if dysbiosis of intracardial microbes has an impact on cardiac function and biology is an intriguing concept and our data suggest that the human heart does harbor microbes in both health and disease and that dysbiosis of these may play a role in the progression of heart disease. However, further in depth studies are necessary to confirm the data as well as elucidating underlying molecular processes, exploring possible human genetic variants of immunological mediators that might influence the susceptibility to microbial signals in relation to heart disease, as well as exploring the possibility of both beneficial and adverse ramifications of cardiac microbes in relation to cardiac function. Such new knowledge and data will frame a novel field of potential therapeutic designs for patients with heart disease, e.g. virulence blockers, antimicrobial peptides, peptidomimetics, antibodies and antisense oligonucleotides.

## Methods

### Human heart biopsies

Transmural biopsies were collected from the right atrium appendage just before venous cannulation for cardiopulmonary bypass in ten patients undergoing coronary artery bypass grafting and ten patients undergoing aortic valve replacement at the Sahlgrenska University Hospital, Gothenburg, Sweden. The tissue was collected in a sterile work area (during surgery), using sterile tubes, reagents and equipment. As a control group, transmural biopsies from explanted hearts were collected from the free wall of the right atrium from five multi-organ donors at the Dept. of Cardiothoracic Surgery at Sahlgrenska University Hospital were used. The donor hearts were not suitable for heart transplantation but explanted for homograft procurement in a GMP-certified facility and used in the present study after the valves were harvested. Organ donors with chronic heart failure were excluded. All tissue was immendiately put in RNA Later that had been specifically checked for contaminants using PCR, which was negative. The samples were then immediately frozen in − 80 degrees. RNA was extracted with RNAse free and sterile laboratory techniques in a laminar flow cabinet. Commercially available right atrium cardiac tissue RNA from another five healthy individuals was also added as control samples (AMS Biotechnology Europe Ltd—Abingdon, U.K.). The study protocol conforms to the ethical guidelines of the 1975 Declaration of Helsinki as reflected in approval by the Ethical Review Board of the University of Gothenburg (reference number 560-12 and 436-15, approved on 2012-10-19 and 2015-06-25, respectively). Written informed consent was obtained from all of the included patients or, for donor hearts, next of kin, stating that their organs could be used for other medical purposes than organ transplantation.

### Multiplex immunoassay analysis of plasma

Plasma EDTA samples from the same patient cohort was collected and analyzed, using Proximity Extension Assay (PEA) technology and the Proseek Multiplex CVD III kit (Olink Proteomics AB, Uppsala, Sweden), according to manufacturers instruction. Analysis of 92 human cardiovascular and inflammatory biomarkers was performed (TATAA Biocenter, Gothenburg Sweden, https://tataa.com). Linear regression calculations with bacterial RNA expression was performed using the GraphPad Prism 7.00 software (San Diego, CA). Local False Discovery Rate (local FDR) values were calculated for the obtained P-values using RStudio version 2022.02.3 with R base version 4.1.2 and the package “qvalue”, treating the analysis for each bacteria separately. P-values < 0.05 with local FDR < 0.05 were considered significant.

### RNA extraction

Total RNA was isolated from heart biopsies with the RNeasy Fibrous Tissue Mini kit (Qiagen, Valencia, CA) as previously described^41^. The tissue was disrupted with 8 mm steal beads using a TissueLyser and treated with Proteinase K for Protease digestion (Qiagen, Valencia, CA).

### Gene expression analysis of Toll-like receptor inflammatory markers

For gene expression analyses of Toll-Like Receptor (TLR)-mediated signal transduction and innate immunity, the human TLR Signaling Pathway RT^2^ Profiler PCR Array (PAHS-018ZA, Qiagen, Valencia, CA) was used as previously described^7^. The qPCR reaction was performed with an ABI 7900 HT fast real time 96 well module (Applied Biosystems, Foster City, CA). All PCR amplification was performed for 40 cycles.

Data were normalized with an automatic selection of genes from the full plate. Qiagen’s Web-based PCR Array Data Analysis Software, available at www.SABiosciences.com/pcrarraydataanalysis.php was used to automatically select an optimal set of internal reference genes for the analysis. The C_T_ values for these genes were then geometrically averaged and used for the delta delta C_T_ calculations. Linear regression calculations with bacterial RNA expression was performed using the GraphPad Prism 7.00 software (San Diego, CA). Local False Discovery Rate (local FDR) values were calculated for the obtained P-values using RStudio, package “qvalue”, treating the analysis for each bacteria separately. P-values < 0.05 with local FDR < 0.05 were considered significant.

### RNA sequencing

RNA sequencing analysis was performed at the Genomics Core Facility at University of Gothenburg, Sweden. All samples were quality checked by the RNA integrity number (RIN) using Tapestation 2200 RNA screenTape (Agilent Technologies, Santa Clara, CA). RIN values ranged between 6.6 and 9.0 for all samples. For a detailed description of the sample preparation, see the “Supplementary materials” and “Methods” section.

Libraries were quantified and normalized with Qubit DNA HS Assay kit (Life Technologies, Carlsbad, CA) and fragment size determined by Tapestation 2200 (Agilent Technologies, Santa Clara, CA). The libraries were pooled together by using the Illumina protocol for pooling and sequenced with NovaSeq 6000 S1 (Illumina, San Diego, CA) for the read length of 2 × 100 bp.

### Metagenomics analysis

The reads of each sample were classified to its best matching organism and taxonomic ID (taxid) using our in-house application Pathogen Research in Clinical Applications (PaRCA), https://github.com/ClinicalGenomicsGBG/PARCA^42^.

The results from PaRCA were used for Differential Expression analysis using DESeq2 where the samples were filtered prior to running DESeq2 to only contain the number of reads of the organisms that were present in at least five of the 30 samples. The normalization was performed using the DESeq2 function called variance stabilizing transformation (VST) that take into account the patient groups so that these do not contribute to the expected variance-mean trend of the experiment (setting blind to false). The results from DESeq2 were filtered for a p-adjusted value of 0.05 and a log2FoldChange ≥ 1 or log2FoldChange ≤ − 1^43^.

Classifications were verified by screening the reads of each organism for their best matching reference, see detailed description in the supplementary materials and “Methods” section.

Multivariate analysis was carried out by principal component analysis (PCA) using SIMCA v.17.0.2 (Umetrics, Umeå, Sweden). To obtain a more normal-like distribution, data were log transformed prior to statistical analysis.

### Genomics analysis – human genes

The raw data was aligned with the human GRCh38.90 reference library from the Ensembl genome browser (https://www.ensembl.org/Homo_sapiens/Info/Index), and the resulting BAM files were used for bioinformatics analysis. Transcripts with counts ≥ 10 in at least nine samples were included in the bioinformatics analysis.

Gene expression analysis and Gene Set Enrichment Analysis (GSEA)^44^ of the gene data presented in Fig. 1d–e were performed with Qlucore Omics Explorer 3.8 (Qlucore AB, Lund, Sweden). The Molecular Signatures Database (MSigDB) was used to obtain gene sets for GSEA, (https://www.gsea-msigdb.org/gsea/msigdb/index.jsp). Levels of significance for differences between group means were determined with ttest or two-way ANOVA followed by Tukey’s multiple comparison tests. A false discovery rate-adjusted p-value < 0.05 was considered significant.

#### Gene and correlation calculations of L. kefiranofaciens (Fig. 2b,c)

All calculations were carried out using RStudio version 2022.02.3 with R base version 4.1.2, with the exception of the GSEA analysis, that in part was performed using the gene set enrichment analysis, GSEA software, and Molecular Signature Database (MSigDB)^44^, http://www.broad.mit.edu/gsea/.

Only genes that had at least 10 counts in at least 8 samples were retained. Normalization was performed using the DESeq2 VST function (see above). For a detailed description of the correlation calculations, see the supplementary material and “Methods” section.

Results were visulalized using the Cytoscape v. 3.9.1 software and the EnrichmentMap app (https://cytoscape.org).

## Supplementary Information

Supplementary Information.

## Data Availability

The datasets generated and/or analysed during the current study are available in the Swedish National Data Service repository, https://snd.gu.se/en, accession link https://doi.org/10.5878/e48r-gn02.

## Abbreviations

AVR: Aortic valve replacement
CABG: Coronary artery bypass grafting
CVD: Cardiovascular disease
DAMP: Damage-associated molecular patterns
GSEA: Gene set enrichment analysis
LPS: Lipopolysaccharide
PAMP: Pathogen-associated microbial patterns
PCA: Principal component analysis
TLR: Toll-like receptor
TMAO: Trimethylamine oxide
