# Supplementary material for: Metagenomic sequencing of human cardiac tissue reveals Microbial RNA which correlates with Toll-like receptor-associated inflammation in patients with heart disease
Source: Sci Rep. 2023 May 15;13:7884. doi: 10.1038/s41598-023-35157-w (PMC10185540; doi:10.1038/s41598-023-35157-w)
Supplement: Supplementary file 1 — Supplementary Information. [file 41598_2023_35157_MOESM1_ESM.pdf]

Metagenomic sequencing of human cardiac tissue reveals Microbial RNA which correlates with Toll-like receptor-associated inflammation in patients with heart disease

Joakim Sandstedt[1], Kristina Vukusic[1], Göran Dellgren[2,3], Anders Jeppsson[2,3], Lillemor Mattsson Hultén[1,4,†] and Victoria Rotter Sopasakis[1,†*]

[1] Department of Clinical Chemistry, Sahlgrenska University Hospital, and Department of Laboratory Medicine, Institute of Biomedicine, Sahlgrenska Academy, University of Gothenburg, Gothenburg, Sweden

[2] Department of Cardiothoracic Surgery, Sahlgrenska University Hospital, Gothenburg, Sweden

[3] Department of Molecular and Clinical Medicine, Institute of Medicine, Sahlgrenska Academy, University of Gothenburg, Gothenburg, Sweden

[4] Wallenberg Laboratory, Department of Molecular and Clinical Medicine, Institute of Medicine, Sahlgrenska Academy, University of Gothenburg, Gothenburg, Sweden

†These authors share senior authorship

Supplementary Figure 1

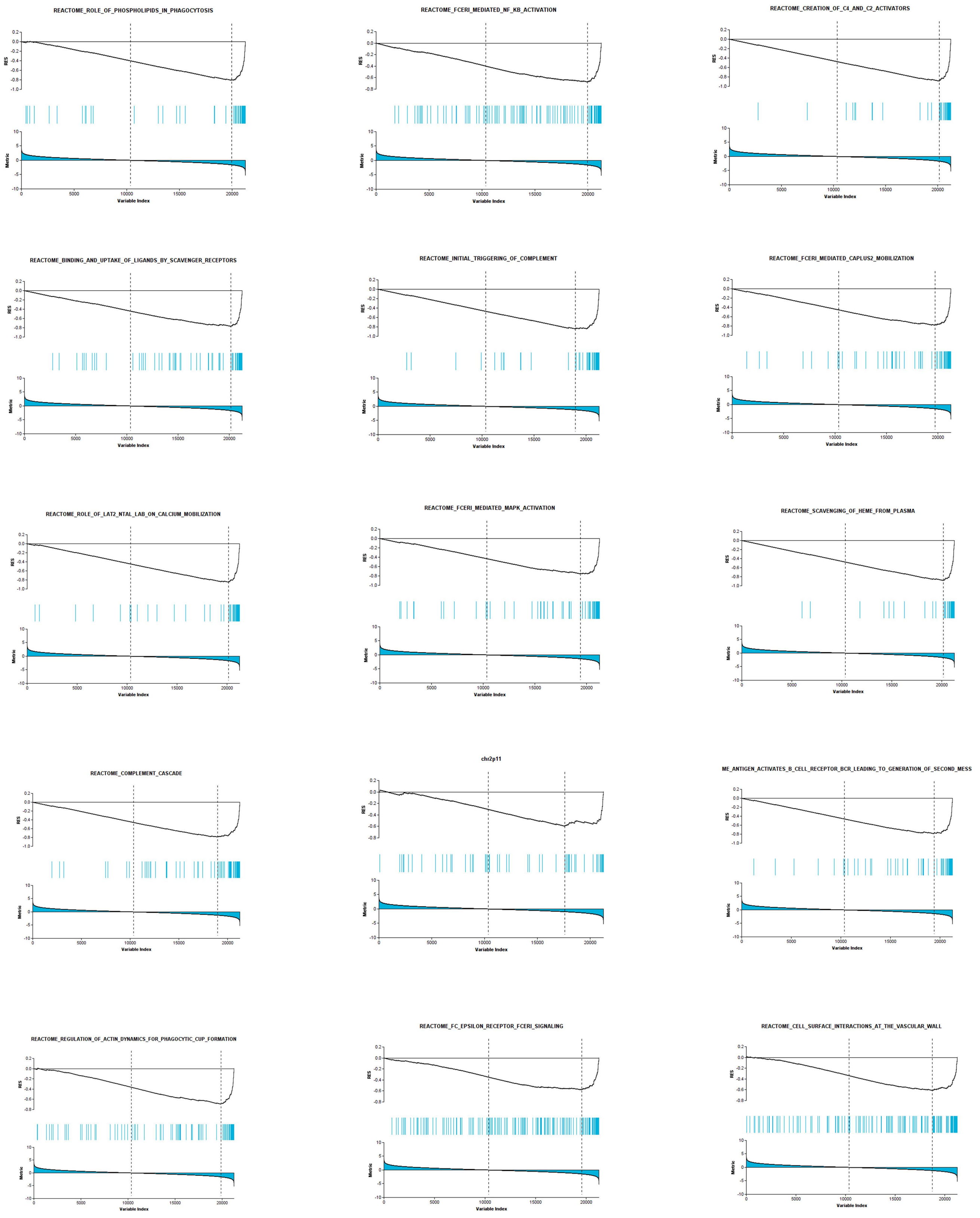

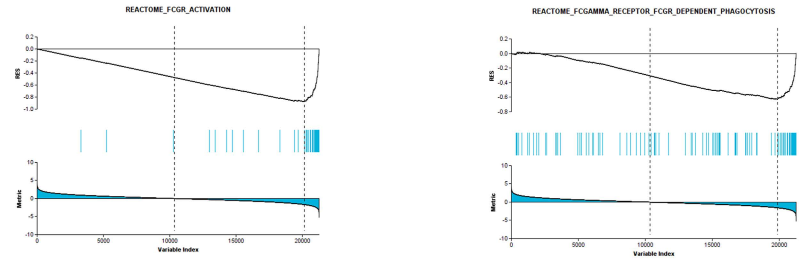

Enrichment score plots of core enrichment genes following Gene Set Enrichment Analaysis (GSEA) of the two subgroups in within the patient group in Fig. 1C.
n=10

Supplementary figure 2

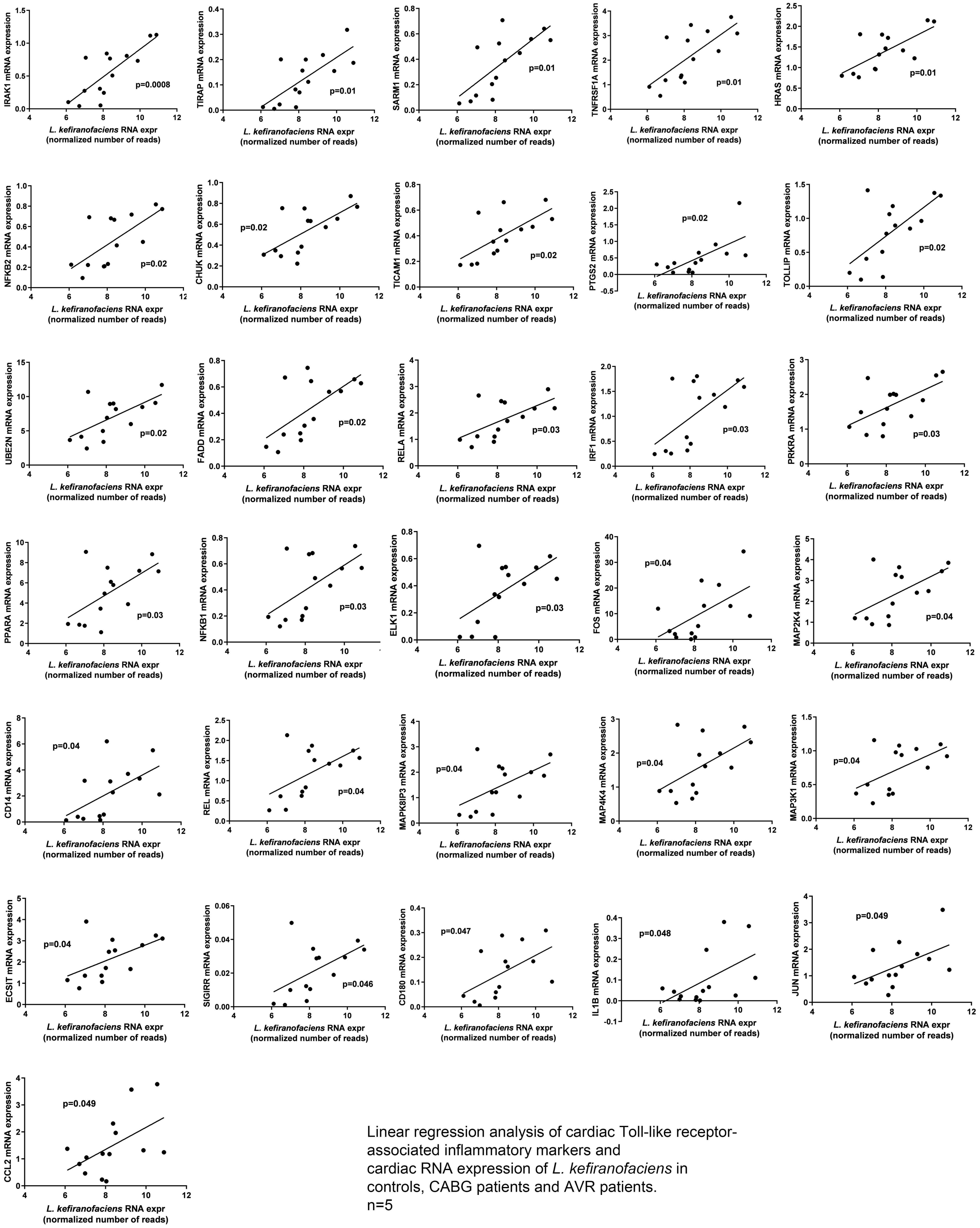

Linear regression analysis of cardiac Toll-like receptor-associated inflammatory markers and cardiac RNA expression of *L. kefiranofaciens* in controls, CABG patients and AVR patients.
n=5

Supplementary Table 1.

Microbial organisms and species detected in human cardiac tissue.

		Relative abundance (variance stabilized counts)		
**Organism**	**Species**	**Controls**	**CABG**	**AVR**
bacteria	*Acidihalobacter prosperus*	7,6±1.9	7,1±0.8	7,2±7.2
bacteria	*Acinetobacter baumannii*	14.3±2.9	14.2±1.3	15±2.2
bacteria	*Acinetobacter sp_p838*	9.0±2.1	9.3±0.5	9.3±0.7
bacteria	*Aerococcus christensenii*	1.6±1.5	2.4±2.1	2.6±2.5
bacteria	*Akkermansia muciniphila*	2.2±2.1	0.8±1.2*10^16	0.8±1.2*10^16
bacteria	*Alcanivorax hongdengensis*	10.9±0.2	10.6±0.2	10.7±0.3
bacteria	*Alcanivorax nanhaiticus*	10.7±0.2	10.4±0.3	10.5±0.3
bacteria	*Alcanivorax sp_n37a*	4.0±3.7	5.4±0.8	5.3±0.7
bacteria	*Alistipes shahii*	2.1±2.3	2.2±1.6	1.3±1.2
bacteria	*Anaerostipes hadrus*	2.1±2.2	2.8±1.8	1.9±1.5
bacteria	*Aquimarina agarivorans*	4.0±2.0	3.8±1.2	3.4±1.9
bacteria	*Bacterium is425*	3.1±2.8	4.2±1.3	4.6±0.7
bacteria	*Bacterium lo352*	3.1±2.9	4.4±0.6	4.3±1.4
bacteria	*Bacterium lo393*	3.0±2.6	2.5±1.5	3.0±1.6
bacteria	*Bacteroides fragilis*	9.0±0.9	8.2±0.2	8.3±0.2
bacteria	*Blautia obeum*	1.8±1.6	2.1±1.7	1.9±1.5
bacteria	*Bordetella bronchiseptica*	8.6±4.1	8.3±4.0	9.2±3.0
bacteria	*Burkholderia multivorans*	2.6±1.8	1.6±1.4	1.3±1.1
bacteria	*Campylobacter jejuni*	8.7±0.4	8.6±0.3	8.8±0.7
bacteria	*Chlamydia psittaci*	4.0±5.2	5.0±5.5	4.0±5.1
bacteria	*Clostridium bolteae*	1.6±1.7	1.8±1.7	1.7±1.5
bacteria	*Clostridium botulinum*	1.7±1.9	2.2±1.5	1.4±1.4
bacteria	*Clostridium perfringens*	1.7±1.7	1.9±2.4	0.8±1.2*10^16
bacteria	*Clostridium sp_l250*	1.4±1.3	3.2±2.6	2.4±2.2
bacteria	*Clostridium sphenoides*	2.1±2.2	1.5±1.2	1.0±0.8
bacteria	*Comamonas testosteroni*	2.5±2.0	4.6±0.4	4.7±0.5
bacteria	*Cutibacterium acnes*	1.9±1.7	3.7±2.1	3.7±1.6
bacteria	*Cyanothece sp_ccy0110*	9.3±0.4	8.9±0.2	9.0±0.2
bacteria	*Dickeya zeae*	4.8±1.5	5.9±0.3	5.7±0.3
bacteria	*Enterobacter cloacae*	8.2±3.9	9.6±0.2	7.9±3.8
bacteria	*Enterobacteriaceae bacterium*	1.3±1.6	2.2±1.5	1.9±1.4
bacteria	*Enterococcus cecorum*	1.1±1.1	4.7±2.5	2.9±3.0
bacteria	*Enterococcus faecalis*	1.3±1.3	2.1±1.8	2.4±2.3
bacteria	*Escherichia coli*	9.2±1.6	10.9±1.0	9.9±3.3
bacteria	*Eubacterium rectale*	1.7±1.6	2.6±2.0	1.9±1.5
bacteria	*Eubacterium ventriosum*	1.8±1.7	1.7±1.6	1.6±1.3
bacteria	*Fusobacterium nucleatum*	3.2±4.0	0.8±1.2*10^16	1.8±1.4
bacteria	*Gardnerella vaginalis*	2.8±2.6	2.9±2.9	2.3±2.1
bacteria	*Gemmata sp_shpl17*	2.5±2.6	2.2±1.9	2.5±1.9
bacteria	*Gordonia amicalis*	8.9±1.5	10.1±0.3	10.1±0.3
bacteria	*Helicobacter pylori*	9.9±0.4	9.6±0.7	9.6±0.5
bacteria	*Klebsiella pneumoniae*	2.9±2.6	4.5±2.0	5.6±0.4
bacteria	*Klebsiella quasipneumoniae*	2.2±2.1	2.3±1.6	2.0±1.6

bacteria	*Lachnospiraceae bacterium_a4*	1.1±1.0	3.0±1.9	2.0±1.7
bacteria	*Lactobacillus acidophilus*	4.6±2.7	8.8±3.2	8.8±1.5
bacteria	*Lactobacillus amylolyticus*	1.4±1.3	3.3±2.4	4.0±2.5
bacteria	*Lactobacillus amylovorus*	3.6±2.3	7.4±1.6	6.5±1.4
bacteria	*Lactobacillus backii*	2.9±2.2	5.5±2.6	6.2±2.8
bacteria	*Lactobacillus brevis*	0.8±1.2*10^16	3.2±2.2	4.1±1.2
bacteria	*Lactobacillus coleohominis*	2.8±2.0	6.1±1.0	6.1±1.4
bacteria	*Lactobacillus crispatus*	6.5±5.3	10.8±7.8	11.6±5.9
bacteria	*Lactobacillus curvatus*	1.2±0.9	2.3±2.0	2.8±2.3
bacteria	*Lactobacillus delbrueckii*	4.5±1.8	7.6±1.6	7.8±1.9
bacteria	*Lactobacillus fermentum*	2.0±2.2	1.8±1.7	1.1±1.2
bacteria	*Lactobacillus fructivorans*	1.4±1.1	4.4±1.5	4.4±1.5
bacteria	*Lactobacillus gallinarum*	2.7±2.4	5.2±3.1	6.3±2.9
bacteria	*Lactobacillus gasseri*	2.6±2.3	3.0±3.1	2.3±3.3
bacteria	*Lactobacillus helveticus*	5.5±2.7	9.9±1.2	9.2±1.3
bacteria	*Lactobacillus iners*	4.7±3.0	5.7±4.4	7.2±4.8
bacteria	*Lactobacillus jensenii*	4.2±2.9	6.2±4.5	6.2±4.4
bacteria	*Lactobacilluss johnsonii*	7.0±2.9	10.2±2.5	10.7±2.8
bacteria	*Lactobacillus kefiranofaciens*	4.6±2.5	9.1±1.1	8.4±1.0
bacteria	*Lactobacillus mali*	2.1±1.3	5.2±0.9	5.1±1.0
bacteria	*Lactobacillus mucosae*	1.0±0.7	3.3±2.2	2.0±2.1
bacteria	*Lactobacillus plantarum*	2.1±1.8	3.4±2.8	4.0±2.3
bacteria	*Lactobacillus reuteri*	1.9±1.8	3.4±2.4	2.6±2.0
bacteria	*Lactobacillus rhamnosus*	2.8±2.3	6.2±2.9	4.7±3.5
bacteria	*Lactobacillus rossiae*	9.4±2.5	14.2±1.9	13.5±2.1
bacteria	*Lactobacillus sakei*	1.2±0.9	2.1±2.1	3.3±1.9
bacteria	*Lactobacillus salivarius*	1.0±0.8	2.1±1.8	3.0±2.2
bacteria	*Lactobacillus sp_7147faa*	1.4±1.4	3.1±3.0	3.9±2.9
bacteria	*Lactobacillus sp_wkb8*	1.3±1.3	2.4±2.1	2.7±2.2
bacteria	*Lactococcus garvieae*	7.6±1.8	8.6±1.2	8.3±0.7
bacteria	*Lysinibacillus saudimassiliensis*	1.3±1.2	2.2±1.9	2.1±2.1
bacteria	*Megasphaera elsdenii*	2.6±2.1	2.2±3.0	1.4±1.9
bacteria	*Micrococcus luteus*	2.7±1.4	2.6±2.0	1.6±1.4
bacteria	*Mycoplasma mycoides*	3.3±3.4	8.2±3.4	7.3±2.3
bacteria	*Neisseria polysaccharea*	6.3±0.2	6.1±0.2	6.2±0.3
bacteria	*Neisseria subflava*	3.9±1.1	3.6±1.5	4.3±0.4
bacteria	*Oscillibacter valericigenes*	2.4±2.7	2.2±1.9	1.9±1.4
bacteria	*Paeniclostridium sordellii*	10.2±2.4	13.3±1.5	13.4±1.0
bacteria	*Pectobacterium carotovorum*	8.1±0.2	7.8±0.2	7.9±0.2
bacteria	*Prevotella intermedia*	2.3±2.4	2.2±1.5	1.0±0.9
bacteria	*Prevotella melaninogenica*	3.1±3.1	1.2±1.2	1.2±1.2
bacteria	*Proteus mirabilis*	1.4±2.1	1.3±1.1	1.4±1.3
bacteria	*Pseudoalteromonas agarivorans*	11.7±0.2	11.5±0.1	11.6±0.2
bacteria	*Pseudoflavonifractor capillosus*	1.8±1.7	2.0±1.6	1.5±1.2
bacteria	*Pseudomonas syringae*	8.1±3.8	11.5±3.2	11.9±1.5
bacteria	*Ralstonia solanacearum*	5.8±5.3	11.0±0.5	10.8±0.7
bacteria	*Rhodococcus opacus*	9.0±1.9	10.0±0.5	9.9±0.5

bacteria	*Roseburia hominis*	1.9±2.0	3.0±2.0	2.5±1.9
bacteria	*Roseburia inulinivorans*	4.2±2.2	2.7±1.8	2.9±1.5
bacteria	*Ruminiclostridium thermocellum*	3.1±2.5	8.0±1.5	6.5±2.5
bacteria	*Ruminococcus gnavus*	1.7±1.8	2.0±1.6	1.9±1.5
bacteria	*Ruminococcus torques*	2.2±2.3	2.6±1.9	2.4±1.7
bacteria	*Salmonella enterica*	2.8±2.2	4.9±2.2	4.8±2.1
bacteria	*Sphingomonas hengshuiensis*	1.7±1.6	2.9±2.8	4.9±1.9
bacteria	*Sphingomonas sp*	2.1±2.1	2.7±1.7	2.4±1.7
bacteria	*Sphingopyxis macrogoltabida*	2.4±3.0	1.9±1.8	2.0±2.0
bacteria	*Staphylococcus aureus*	7.6±3.6	12.2±0.3	12.2±0.4
bacteria	*Stenotrophomonas maltophilia*	1.7±2.1	1.1±0.9	1.4±1.3
bacteria	*Streptococcus agalactiae*	8.2±6.4	8.1±6.3	6.8±6.4
bacteria	*Streptococcus australis*	1.9±1.9	1.6±1.7	2.1±1.4
bacteria	*Streptococcus equi*	1.8±1.7	1.8±1.7	1.4±1.4
bacteria	*Streptococcus mutans*	5.3±2.0	4.6±1.4	4.8±1.6
bacteria	*Streptococcus pneumoniae*	8.3±0.1	7.9±0.3	7.9±0.2
bacteria	*Streptococcus pyogenes*	2.0±1.6	2.2±2.1	2.1±1.4
bacteria	*Tyzzerella nexilis*	1,7±1.0	4.1±0.4	3.5±1.0
bacteria	*uncultured bacterium*	6.0±1.0	4.8±0.7	4.5±0.5
bacteria	*uncultured Bacterium_sat21c112b*	2.1±2.1	6.1±6.9	4.8±5.7
bacteria	*uncultured Lachnospiraceae_bacterium*	1.8±1.7	2.7±1.7	2.3±1.6
bacteria	*uncultured Lactobacillus_sp*	3.8±2.5	7.1±0.9	6.9±0.9
bacteria	*uncultured Megasphaera_sp*	1.3±1.2	1.9±2.5	1.3±1.6
bacteria	*uncultured Oscillibacter_sp*	1.9±1.9	1.3±1.2	0.8±1.2*10^16
bacteria	*uncultured Sphingomonas_sp*	1.7±1.6	1.6±1.3	2.8±1.9
bacteria	*uncultured Streptococcus_sp*	1.4±1.0	3.4±3.1	3.3±3.3
bacteria	*Waddlia chondrophila*	1.2±0.8	2.5±2.0	1.7±2.2
bacteria	*Veillonella parvula*	3.1±3.3	1.8±1.7	1.7±1.5
bacteria	*Vibrio vulnificus*	6.9±3.4	6.2±2.9	6.2±2.9
virus	*Bean 58058 virus*	13.6±0.3	13.9±0.2	13.7±0.4
virus	*Chrysochromulina ericina virus*	1.3±1.1	4.7±1.6	3.7±1.1
virus	*Guanarito mammarenavirus*	1.8±1.6	1.6±1.7	1.1±1.1
virus	*Human endogenous retrovirus_k*	10.8±0.3	11.4±0.2	11.4±0.2
virus	*Murine leukemia virus*	1.0±0.9	3.4±1.5	2.7±1.7
virus	*Salmonella virus sp6*	2.3±3.3	4.0±5.5	2.7±4.3
virus	*Shamonda orthobunyavirus*	2.8±2.3	5.1±0.6	5.3±0.4

Supplementary Table 2.

Linear regression analysis of cardiovascular plasma biomarkers and cardiac atrial bacterial RNA expression in CABG and AVR patients (combined), n=20

L. kefiranofaciens		
**Gene**	**p-value**	**FDR-adjusted p-value**
COL1A1	0.005	0.30
CCL24	0.01	0.40
ICAM2	0.01	0.44
MEPE	0.02	0.49
EGFR	0.03	0.54
AP_N	0.04	0.58
CPA1	0.07	0.62
IL2RA	0.08	0.63
CHI3L1	0.08	0.63
IL1RT2	0.12	0.67
Gal4	0.12	0.67
PI3	0.12	0.67
TNFR2	0.12	0.67
CPB1	0.14	0.69
BLM hydrolase	0.14	0.69
SELE	0.14	0.69
CSTB	0.16	0.70
IGFBP1	0.16	0.71
CDH5	0.16	0.71
PON3	0.17	0.72
ITGB2	0.17	0.72
PSP_D	0.18	0.73
CHIT1	0.19	0.74
PDGF subunit A	0.20	0.75
CNTN1	0.22	0.77
JAMA	0.23	0.77
MCP1	0.24	0.78
MMP3	0.25	0.80
CASP3	0.26	0.81
Ep_CAM	0.28	0.82
GRN	0.28	0.83
TNFRSF14	0.29	0.83
TNFRSF10C	0.29	0.83
PAI	0.29	0.83
ST2	0.30	0.84
U_PAR	0.30	0.84
12-HETE	0.32	0.85
PGLYRRP1	0.34	0.87
TNFR1	0.35	0.88
vWF	0.35	0.88
Notch3	0.35	0.88
AXL	0.39	0.90

TLT2	0.41	0.91
SELP	0.42	0.92
MB	0.43	0.92
SCGB3A2	0.44	0.92
GDF15	0.47	0.93
Gal3	0.48	0.93
LTBR	0.48	0.93
IL1RT1	0.51	0.93
OPN	0.52	0.93
SHPS1	0.52	0.93
tPA	0.54	0.93
MMP2	0.54	0.93
5-HETE	0.57	0.93
DLK1	0.58	0.93
TR	0.59	0.93
PECAM1	0.59	0.93
IGFBP7	0.64	0.93
FAS	0.64	0.93
TNFSF13B	0.64	0.93
PLC	0.65	0.93
PRTN3	0.65	0.93
CCL15	0.66	0.93
LDLR	0.68	0.93
NTproBNP	0.70	0.93
OPG	0.73	0.93
CD163	0.73	0.93
ALCAM	0.73	0.93
IL18BP	0.75	0.93
TFF3	0.75	0.93
CXCL16	0.76	0.93
RARRES2	0.76	0.93
uPA	0.77	0.93
TLT2	0.78	0.93
GP6	0.79	0.93
15-HETE	0.79	0.93
MMP9	0.79	0.93
CD93	0.80	0.93
MPO	0.81	0.93
CTSD	0.82	0.93
IL17RA	0.82	0.93
TIMP4	0.85	0.93
IL6RA	0.86	0.93
PCSK9	0.87	0.93
CCL16	0.87	0.93
AZU1	0.87	0.93
KLK6	0.89	0.93
TR_AP	0.91	0.93

SPON1	0.91	0.93
RETN	0.92	0.93
CTSZ	0.93	0.93
EPHB4	0.96	0.93
IGFBP2	0.97	0.93
FABP4	0.99	0.93
L. amylovorus		
**Gene**	**p-value**	**FDR-adjusted p-value**
AP_N	0.01	0.49
EGFR	0.02	0.51
IL1RT1	0.02	0.52
PAI	0.04	0.54
IL1RT2	0.07	0.55
COL1A1	0.07	0.55
CHI3L1	0.07	0.55
MEPE	0.08	0.55
CDH5	0.09	0.55
BLM hydrolase	0.10	0.55
AXL	0.11	0.56
PI3	0.12	0.56
DLK1	0.14	0.58
tPA	0.15	0.58
IL2RA	0.15	0.58
LDLR	0.16	0.59
TLT2	0.17	0.60
PDGF subunit A	0.17	0.60
Gal4	0.18	0.60
ICAM2	0.19	0.61
GRN	0.19	0.61
MCP1	0.19	0.61
CPA1	0.20	0.62
GP6	0.20	0.62
IGFBP7	0.21	0.62
IL18BP	0.21	0.63
U_PAR	0.21	0.63
ALCAM	0.22	0.63
PSP_D	0.23	0.64
CCL24	0.23	0.64
IGFBP1	0.24	0.65
PON3	0.24	0.65
GDF15	0.24	0.65
CSTB	0.26	0.67
PCSK9	0.27	0.67
CD163	0.28	0.68
PGLYRRP1	0.29	0.69
PECAM1	0.29	0.69

OPG	0.30	0.70
EPHB4	0.32	0.72
IL6RA	0.36	0.74
ITGB2	0.39	0.76
CHIT1	0.40	0.77
Ep_CAM	0.40	0.77
JAMA	0.41	0.77
IL17RA	0.41	0.78
uPA	0.42	0.78
OPN	0.43	0.78
ST2	0.43	0.78
NTproBNP	0.45	0.79
SHPS1	0.45	0.80
TR_AP	0.46	0.80
CASP3	0.46	0.80
MMP9	0.47	0.80
12-HETE	0.48	0.81
CPB1	0.51	0.82
RETN	0.53	0.83
SPON1	0.54	0.83
CD93	0.55	0.83
TNFRSF10C	0.55	0.83
Gal3	0.56	0.84
15-HETE	0.57	0.84
PLC	0.58	0.84
TIMP4	0.58	0.84
TFF3	0.61	0.85
PRTN3	0.62	0.85
MB	0.62	0.85
SELE	0.63	0.85
SCGB3A2	0.64	0.85
CNTN1	0.65	0.86
CCL15	0.67	0.86
TLT2	0.68	0.86
MPO	0.69	0.87
Notch3	0.69	0.87
FABP4	0.72	0.87
TR	0.72	0.87
KLK6	0.77	0.88
5-HETE	0.77	0.88
SELP	0.78	0.88
CTSD	0.78	0.88
CTSZ	0.78	0.88
RARRES2	0.78	0.88
TNFR2	0.80	0.89
TNFSF13B	0.81	0.89
MMP2	0.81	0.89

LTBR	0.83	0.89
vWF	0.83	0.89
MMP3	0.91	0.89
CXCL16	0.91	0.89
IGFBP2	0.92	0.89
FAS	0.93	0.89
TNFR1	0.95	0.89
AZU1	0.96	0.89
CCL16	0.96	0.89
TNFRSF14	0.96	0.89
L. helveticus		
**Gene**	**p-value**	**FDR-adjusted p-value**
AP_N	0.02	0.49
COL1A1	0.02	0.49
CHI3L1	0.03	0.52
MEPE	0.04	0.54
EGFR	0.05	0.55
PAI	0.05	0.56
PECAM1	0.06	0.57
IL1RT1	0.06	0.59
U_PAR	0.06	0.59
IL2RA	0.08	0.61
AXL	0.09	0.64
IL1RT2	0.10	0.67
PON3	0.12	0.69
BLM hydrolase	0.13	0.70
PSP_D	0.13	0.70
CSTB	0.14	0.73
PDGF subunit A	0.14	0.73
CDH5	0.14	0.73
tPA	0.15	0.74
CPA1	0.15	0.74
DLK1	0.15	0.75
ICAM2	0.17	0.78
IGFBP7	0.20	0.83
MCP1	0.20	0.83
GDF15	0.21	0.84
SHPS1	0.21	0.85
Gal4	0.22	0.86
TLT2	0.23	0.88
CCL24	0.23	0.88
PI3	0.24	0.89
GRN	0.24	0.89
ITGB2	0.25	0.90
PCSK9	0.25	0.91
OPG	0.26	0.92

IL6RA	0.26	0.93
LDLR	0.26	0.93
CHIT1	0.27	0.94
uPA	0.28	0.96
GP6	0.31	1
ALCAM	0.33	1
CD163	0.33	1
TLT2	0.34	1
IGFBP1	0.34	1
PRTN3	0.35	1
CPB1	0.35	1
IL17RA	0.36	1
12-HETE	0.37	1
ST2	0.37	1
MPO	0.40	1
IL18BP	0.41	1
NTproBNP	0.44	1
PGLYRRP1	0.44	1
Ep_CAM	0.45	1
EPHB4	0.45	1
SELP	0.47	1
CNTN1	0.51	1
TNFR2	0.59	1
RARRES2	0.61	1
TFF3	0.63	1
AZU1	0.64	1
Notch3	0.66	1
CCL15	0.66	1
SELE	0.68	1
TIMP4	0.70	1
15-HETE	0.70	1
JAMA	0.71	1
PLC	0.73	1
FABP4	0.75	1
MMP2	0.75	1
CTSD	0.75	1
LTBR	0.75	1
TNFRSF10C	0.76	1
CASP3	0.76	1
CD93	0.76	1
OPN	0.77	1
IGFBP2	0.79	1
MB	0.79	1
TNFR1	0.79	1
TNFSF13B	0.80	1
TNFRSF14	0.81	1
SPON1	0.82	1

KLK6	0.82	1
Gal3	0.83	1
CXCL16	0.85	1
TR_AP	0.85	1
SCGB3A2	0.85	1
RETN	0.91	1
MMP3	0.92	1
FAS	0.94	1
MMP9	0.94	1
CCL16	0.94	1
TR	0.98	1
CTSZ	0.98	1
vWF	0.99	1
5-HETE	1.00	1

L. backii		
**Gene**	**p-value**	**FDR-adjusted p-value**
MMP9	0.01	1
IL18BP	0.04	1
MB	0.04	1
IL1RT1	0.07	1
LDLR	0.08	1
TLT2	0.08	1
PAI	0.09	1
PON3	0.09	1
PI3	0.12	1
CTSD	0.13	1
GP6	0.13	1
OPN	0.13	1
AZU1	0.14	1
RETN	0.16	1
TR_AP	0.16	1
PCSK9	0.17	1
DLK1	0.19	1
IGFBP2	0.20	1
IGFBP1	0.22	1
NTproBNP	0.23	1
EPHB4	0.23	1
OPG	0.27	1
AP_N	0.28	1
vWF	0.30	1
TNFRSF10C	0.30	1
TLT2	0.31	1
TIMP4	0.31	1
CD93	0.31	1
CNTN1	0.31	1
CHIT1	0.34	1

tPA	0.34	1
PLC	0.34	1
MCP1	0.35	1
Notch3	0.35	1
5-HETE	0.37	1
EGFR	0.37	1
CPB1	0.38	1
TR	0.38	1
JAMA	0.38	1
PRTN3	0.40	1
GDF15	0.40	1
PDGF subunit A	0.41	1
TNFR2	0.43	1
PECAM1	0.43	1
TNFRSF14	0.43	1
ALCAM	0.44	1
CDH5	0.45	1
SPON1	0.47	1
PGLYRRP1	0.47	1
ST2	0.48	1
KLK6	0.49	1
MMP2	0.49	1
CTSZ	0.50	1
TNFR1	0.51	1
15-HETE	0.53	1
uPA	0.56	1
MPO	0.58	1
Gal3	0.58	1
CASP3	0.58	1
AXL	0.59	1
SELP	0.60	1
SCGB3A2	0.61	1
CHI3L1	0.64	1
Gal4	0.66	1
SHPS1	0.66	1
IL17RA	0.66	1
U_PAR	0.69	1
FABP4	0.69	1
CCL24	0.70	1
TNFSF13B	0.70	1
COL1A1	0.70	1
MMP3	0.71	1
CSTB	0.71	1
FAS	0.72	1
CXCL16	0.73	1
IL1RT2	0.73	1
GRN	0.73	1

ICAM2	0.73	1
CCL15	0.76	1
CD163	0.78	1
TFF3	0.80	1
CCL16	0.81	1
CPA1	0.84	1
ITGB2	0.85	1
12-HETE	0.86	1
SELE	0.88	1
PSP_D	0.91	1
RARRES2	0.92	1
IL2RA	0.94	1
LTBR	0.95	1
IGFBP7	0.95	1
IL6RA	0.96	1
MEPE	0.99	1
BLM hydrolase	0.99	1
Ep_CAM	0.99	1
L. johnsonii		
**Gene**	**p-value**	**FDR-adjusted p-value**
MMP9	0.02	0.76
PAI	0.02	0.78
IL1RT1	0.08	0.79
LDLR	0.08	0.79
PCSK9	0.09	0.79
AZU1	0.10	0.79
IL18BP	0.15	0.79
TR_AP	0.16	0.79
AP_N	0.17	0.79
PLC	0.21	0.79
RETN	0.21	0.79
tPA	0.22	0.79
MB	0.23	0.79
OPG	0.23	0.79
CTSD	0.23	0.79
GP6	0.24	0.79
vWF	0.26	0.79
MCP1	0.26	0.79
PON3	0.26	0.79
5-HETE	0.28	0.79
GDF15	0.28	0.79
SPON1	0.31	0.79
CNTN1	0.31	0.79
TIMP4	0.32	0.79
EGFR	0.32	0.79
TLT2	0.33	0.79

EPHB4	0.35	0.79
PI3	0.36	0.79
CPB1	0.36	0.79
DLK1	0.37	0.79
OPN	0.37	0.79
PDGF subunit A	0.37	0.79
NTproBNP	0.38	0.79
uPA	0.42	0.79
MPO	0.42	0.79
15-HETE	0.42	0.79
JAMA	0.44	0.79
PECAM1	0.45	0.79
FABP4	0.49	0.79
ALCAM	0.49	0.79
PRTN3	0.49	0.79
TLT2	0.49	0.79
Notch3	0.49	0.79
IGFBP2	0.50	0.79
IGFBP1	0.52	0.79
CD93	0.52	0.79
PGLYRRP1	0.53	0.79
MMP3	0.53	0.79
MMP2	0.54	0.79
IL2RA	0.55	0.79
CASP3	0.55	0.79
CHIT1	0.55	0.79
TNFR2	0.58	0.79
SELP	0.58	0.79
CCL24	0.59	0.79
12-HETE	0.61	0.79
ITGB2	0.62	0.79
FAS	0.62	0.79
IL1RT2	0.63	0.79
ICAM2	0.65	0.79
RARRES2	0.65	0.79
CHI3L1	0.66	0.79
CDH5	0.66	0.79
COL1A1	0.68	0.79
TNFRSF14	0.70	0.79
LTBR	0.71	0.79
GRN	0.71	0.79
TNFSF13B	0.71	0.79
TR	0.71	0.79
SELE	0.71	0.79
TNFRSF10C	0.71	0.79
SCGB3A2	0.74	0.79
CCL15	0.74	0.79

Gal3	0.75	0.79
CCL16	0.75	0.79
ST2	0.75	0.79
BLM hydrolase	0.75	0.79
CTSZ	0.76	0.79
Gal4	0.76	0.79
SHPS1	0.76	0.79
AXL	0.77	0.79
Ep_CAM	0.80	0.79
CPA1	0.81	0.79
IL6RA	0.83	0.79
IGFBP7	0.83	0.79
CXCL16	0.86	0.79
MEPE	0.86	0.79
CD163	0.88	0.79
TNFR1	0.88	0.79
CSTB	0.89	0.79
PSP_D	0.91	0.79
KLK6	0.92	0.79
TFF3	0.93	0.79
U_PAR	0.95	0.79
IL17RA	0.99	0.86

Supplementary materials and methods

Sample preparation for RNA sequencing

A total of 10 µl (~1 µg) RNA from each sample was used for library preparation. Directly after depletion, a cleanup step was performed using 110 ul of the RNAClean XP beads (Beckman Coulter, USA) for each sample. The fragmentation step was performed for 8 minutes. 12 PCR cycles were run for all samples.

Libraries were quantified and normalized with Qubit DNA HS Assay kit (Life Technologies, Carlsbad, CA) and fragment size determined by Tapestation 2200 (Agilent Technologies, Santa Clara, CA). The libraries were pooled together by using the Illumina protocol for pooling and sequenced with NovaSeq 6000 S1 (Illumina, San Diego, CA) for the read length of 2x100 bp.

Metagenomics analysis

Verifying classifications

Organisms that were differentially expressed were screened for their best matching reference respectively according to the following procedure: All genomes and sequences including the taxid that the organism was classified to by PaRCA were downloaded using Taxonkit together with Ncbi-genome-download. The reads of each organism were screened for their best matching reference one sample at a time using Mash against these downloaded genomes and sequences including the taxid that PaRCA had classified the reads to. Mash checks for containment of the sample in the reference sequence. The best reference was voted to be the reference that was the best match for most of the samples, one taxid at the time. Reads classified to a taxid across all samples were merged for taxids where it was not possible to find a best reference using a vote from all samples respectively. The merged samples were screened using Mash against the taxids and including the

taxid that PaRCA had classified the reads to (1, 2). Mapping was performed by merging all reads classified to the same taxid across all samples and thereby using Bowtie2 with the settings --end-to-end --very-sensitive --no-unal -k 1 for the mapping (3).

Correlation analysis of L. kefiranofaciens RNA expression with human gene sets (Fig. 2B-C)

Correlation between expression of *L. kefiranofaciens* and gene expression was calculated using the cor.test function in R, Pearson's method. Correlation coefficient (r) and p-value for the correlation was retrieved. For subsequent GSEA analysis with phenotypic randomization, sample identity was first randomly reshuffled for the expression value of the *L. kefiranofaciens*, after which correlation was calculated as described above. 1500 randomizations was used. Results from all correlations of phenotypically randomized samples were stored for subsequent analyses.

For GSEA, a rank was calculated for both the "real" correlation results as well as phenotypic randomizations, using sign(cor)* -log10(P), where P is the P-value of the correlations. GSEA was performed on all ranks using the GSEA software (from R, using command line functionality in the GSEA software), gene set "c2.all.v7.2.symbols.gmt". P-values were adjusted for multiple testing using the p.adjust function in R, "fdr" option.

1. Blin K, Shaw S, Kautsar SA, Medema MH, and Weber T. The antiSMASH database version 3: increased taxonomic coverage and new query features for modular enzymes. *Nucleic acids research.* 2021;49(D1):D639-D43.
2. Shen W, and Xiong J. TaxonKit: a cross-platform and efficient NCBI taxonomy toolkit. *Biorxiv.* 2019:513523.
3. Langmead B, and Salzberg SL. Fast gapped-read alignment with Bowtie 2. *Nature methods.* 2012;9(4):357-9.
